# The Treatment of Rupture of the Uterus

**Published:** 1880-12

**Authors:** 


THE TREATMENT OF RUPTURE OF THE UTERUS.

In a recent number of the *Zeitschrift fuer Gynœkologie*, Dr. Frommel, of Berlin, reported a case of perforating uterine rupture, which had been successfully treated by establishing abdominal drainage and irrigating the peritoneal cavity with carbolized water. The author now reports two new cases, which, under similar treatment, likewise recovered. The first case was that of a small woman, aged forty, who had given birth to twelve children. Three of them had been delivered by artificial means. During her last confinement the labor-pains suddenly ceased, and a subsequent very painful examination showed that the child had partially escaped from the uterus into the abdominal cavity. With some difficulty a dead fœtus was extracted, and an examination now revealed a transverse rupture of uterus at its lower segment near the cervical junction. The uterus was almost completely torn off, only anteriorly and to the left a bridge of tissue existed, measuring about five to six centimetres. The peritoneal coat, however, was not ruptured, but had been stripped off by the protruding fœtus. The entire cavity was cleansed by a copious irrigation of a warm aqueous solution of carbolic acid (two per cent.), and a thick drainage-tube was then inserted. Ice was applied over the abdomen. The patient never vomited, her pulse was always good, her temperature never rose above 100.4° F. Only one intra-uturine injection was practiced through drainage-tube, which was removed as soon as the discharge ceased. This occurred on the twenty-sixth day after the accident. Two days later the patient was able to leave her bed, and shortly afterward was discharged in apparently perfect health.

Case II, was that of a woman, thirty years old, who in her youth had suffered from rachitis. She had had three confinements, each with tedious labor. Uterine rupture took place, perhaps in consequence of the administration by a midwife of four ergot powders. Prof. Schröder succeeded in extracting the dead child, which, together with the placenta, was found lodged in the abdominal cavity. The treatment was in all respects similar to that adopted in the first case, with the difference that, in the present instance, carbolized injections were made through the drainage-tube every time the temperature rose above 100.4° F., which occurred on several evenings.

In his remarks on the successful termination of these cases, the author observes that this plan of treatment is much to be preferred to laparotomy. But he admits that it is applicable only to cases in which septic infection has not already taken place, though he adds that laparotomy under such circumstances would also necessitate a bad prognosis.—*Centralblatt fuer Gynæk.*, August 28, 1880.—*New York Medical Record.*

